# Gaps in trait data and conservation impediments from the Raunkiæran shortfall in a megadiverse nation

**DOI:** 10.1016/j.isci.2025.114000

**Published:** 2025-11-10

**Authors:** Krizler C. Tanalgo

**Affiliations:** 1 Ecology and Biodiversity Synthesis Group, Ecology and Conservation Research Laboratory (Eco/Con Lab), Department of Biological Sciences, College of Science and Mathematics, University of Southern Mindanao, Kabacan 9407, Philippines; 2 Eco/Con Lab Biodiversity Synthesis+ Centre, University of Southern Mindanao, Kabacan 9407, Philippines; 3 The Graduate School of the University of Southern Mindanao, Kabacan 9407, Philippines

**Keywords:** Ecology, Evolutionary ecology, Nature conservation

## Abstract

Global biodiversity loss is accelerating, and data access is crucial for supporting research and decision-making to prevent species decline. Research efforts to understand factors driving species vulnerability remain disproportionately distributed across the tree of life. I examined life-history trait data gaps of Philippine terrestrial vertebrates by analyzing differences across taxonomic classes. The analysis showed that herptiles remain understudied compared to birds and mammals, with sampling biased toward larger, widespread, and less threatened species. I argue that these biases and data gaps can lead to misleading generalizations, hindering effective trait-based conservation strategies by limiting data needed to predict at-risk populations. This study highlights challenges faced by megadiverse nations like the Philippines, where bureaucracy, funding limitations, and competing priorities constrain trait-based biodiversity research. Addressing these gaps requires integrated strategies combining improved data collection, research investments, FAIR data sharing, and capacity-building to emphasize trait-based conservation in preventing biodiversity losses.

## Highlights

• Raunkiæran shortfall remains widespread in megadiverse developing nations
• Herptiles have lower trait coverage and completeness than birds and mammals
• Larger, non-endemic, and less threatened species showed better trait sampling
• Research effort and observations increase trait data completeness

## Graphical abstract

**Figure:**
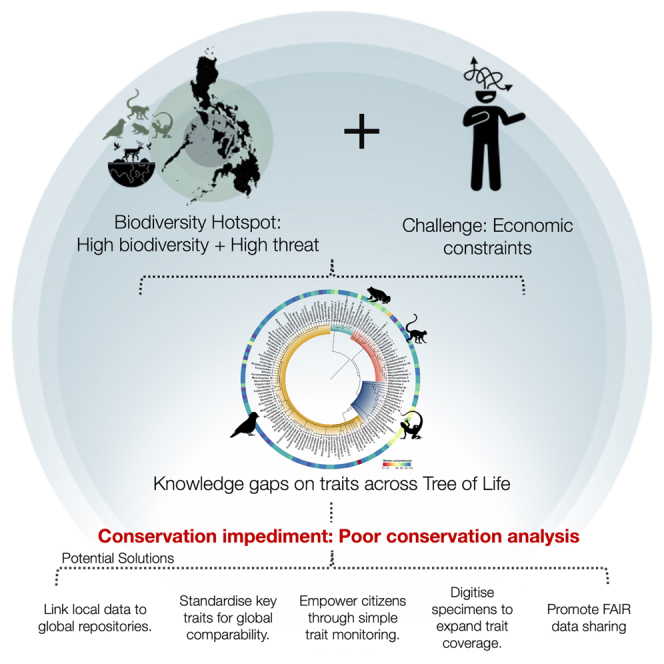

## Introduction

Current species extinction occurs at a rate far exceeding the historical baselines.^1^^,^^2^ Recent estimates suggest that extinction rates are at least 100–1,000 times higher than the natural background rate, signaling the onset of the sixth mass extinction.^1^^,^^2^^,^^3^^,^^4^^,^^5^^,^^6^ Species are disappearing before they can be described, and many decline silently without comprehensive assessment, documentation, and monitoring.^7^ This crisis is not limited to large iconic animals or tropical rainforests; it spans all taxonomic groups and ecosystems, from amphibians and insects to coral reefs and freshwater habitats.^8^^,^^9^^,^^10^ The drivers of extinction are multifaceted, synergistic, and often disproportionately dependent on species and ecological traits.^11^^,^^12^

Danish botanist Christen Raunkiær emphasized that trait-based classification of species provides greater predictive power than relying on taxonomic relationships alone, enabling a better understanding of which populations are at higher risk and how they respond to environmental changes.^13^^,^^14^ In modern ecological studies, many ecologists and conservation biologists rely on trait-based approaches to comprehend patterns of biodiversity distribution and extinction risk through measurable species characteristics.^15^^,^^16^^,^^17^ By examining the relationships between species traits and their vulnerability to various threats, conservation biologists can identify populations at greater risk and implement preventive measures to mitigate future losses.^11^^,^^12^ For instance, species with larger body sizes, such as megafauna, are often at a higher risk of extinction because of their slower reproductive rates, greater resource requirements, and increased susceptibility to human impacts, such as hunting and habitat loss.^18^^,^^19^ Similarly, species with specialized diets or habitats are more vulnerable to environmental changes because of their lower adaptability to altered conditions and stochastic events^18^ (Table 1). Thus, understanding the mechanisms and predictors of extinction risk in the rapidly changing human-dominated era is critical for preventing biodiversity loss and designing effective conservation strategies.^38^^,^^39^

**Table 1:** Simple guide to life-history and their implications for species extinction risk for terrestrial vertebrates

1. Body mass or size: larger-bodied species face higher extinction risk due to greater energy needs, slower reproduction, and increased vulnerability to hunting and habitat fragmentation.^12^^,^^18^^,^^20^ In contrast, smaller species may be less vulnerable to exploitation but risk extinction through habitat specialization and sensitivity to environmental fluctuations.^10^^,^^21^ 2. Habitat breadth: species with narrow habitat requirements are more vulnerable to extinction due to limited adaptability.^11^ Generalist species with broad habitat tolerance are resilient to environmental changes. 3. Lower elevation limits: species in lower elevations face increased human activities, such as agriculture and urbanization, which increase their extinction risk.^22^^,^^23^ Furthermore, climate change can force them to higher elevations, leading to habitat compression or loss.^24^ 4. Upper elevation limits: species at high elevations are particularly vulnerable to climate change as warming temperatures push them upward, potentially causing habitat loss at mountain tops, a phenomenon known as “mountain-top extinction.”^25^^,^^26^^,^^27^^,^^28^ 5. Activity time (diel time): nocturnal species face risks from artificial light pollution, which disrupts natural behaviors and prey-predator dynamics.^29^ Diurnal species face more direct human disturbance through hunting and habitat destruction, increasing extinction risk. 6. Trophic levels: species at higher trophic levels, such as top predators, are more vulnerable to extinction because they rely on stable prey populations, face bioaccumulation of toxins, and experience human-wildlife conflict.^30^^,^^31^^,^^32^ In contrast, species at lower trophic levels, such as herbivores, may be more resilient but face threats from habitat loss and climate change. Herbivores may also be at higher risk due to their body size and risk of hunting.^32^ 7. Litter or clutch size: species with smaller litter sizes have lower reproductive rates, making them susceptible to population declines. Species with larger clutch sizes may be more resilient to environmental fluctuations, which could indicate higher offspring mortality.^21^ 8. Longevity: long-lived species face a higher extinction risk from slow population recovery and delayed reproductive maturity.^33^^,^^34^ Short-lived species may be more resilient through faster reproduction but remain sensitive to rapid environmental changes.^35^ 9. Migration patterns: migratory species face a higher extinction risk by depending on multiple habitats, making them susceptible to habitat loss and environmental changes across their range.^36^ Non-migratory species may be more resilient but remain vulnerable if their local habitat is disrupted. 10. Use of artificial habitats: species that can utilize artificial habitats may be more resilient to habitat loss caused by human activities. However, reliance on artificial habitats can expose them to novel threats, including pollution and predation by invasive species.^29^^,^^37^

Despite the promise of trait-based conservation as a cornerstone of modern biodiversity conservation and management,^40^ collecting and assembling trait data essential for predicting species loss and supporting conservation decisions remains a challenge.^41^ Trait-based conservation relies heavily on comprehensive species trait data; however, these data are incomplete, and coverage for a wide range of taxa is often poor.^39^^,^^42^ The Raunkiæran shortfall or persistent gaps in trait data pose a significant barrier to effective conservation planning, especially in biodiversity-rich areas where the extinction risk and biodiversity loss are also the highest.^1^^,^^2^ Moreover, the relationship between traits and extinction risk is context dependent and can vary significantly across ecosystems, limiting the applicability of universal models.^11^^,^^12^^,^^39^ These challenges are particularly acute in highly diverse but understudied regions, where critical data gaps hinder robust analysis and conservation prioritization and, precisely, where such efforts are urgently needed.^43^

For instance, a recent analysis of extinction risk in Philippine terrestrial vertebrates estimated that at least 15%–23% of Philippine terrestrial vertebrates are threatened with extinction due to habitat loss, overexploitation, and the accelerating impacts of climate change.^44^^,^^45^ In addition, intrinsic variables (e.g., body size, endemism, and trophic levels) are significant drivers of population loss.^46^ However, it is argued that the analysis was potentially limited by the lack of available trait data, leaving the interplay of other traits with extinction risk inadequate.^46^ Building on the work of Tanalgo et al.^46^ and Etard et al.,^39^ I aimed to understand the country-specific pattern and highlight the persistence of the Raunkiæran shortfall in a developing and megadiverse country with numerous taxonomically understudied species,^47^^,^^48^^,^^49^ as a case study, and tackle their conservation relevance. While only drawing from a country-specific perspective, it targets to provide more detailed support for understanding the research limitations faced by scientists, offering a crucial context for addressing data shortfalls and informing practical and effective conservation and research strategies.^50^ I argue that limitations in funding support, a shortage of trained personnel, research bureaucracy, and inadequate research infrastructure may constrain the country’s capacity for comprehensive data collection and further integration to analysis.^51^^,^^52^^,^^53^

In this study, I accounted for trait completeness and coverage of ten commonly known traits across terrestrial vertebrate taxa, leveraging the available global databases. Given their higher detectability and relative ease of sampling, I hypothesized that endotherms (mammals and birds) would exhibit greater data completeness than ectotherms (amphibians and reptiles). I posit that variation in trait completeness is related to range size, species body size, extinction risk, country endemism, year of discovery, research effort, and the number of species threats. Specifically, I expected trait completeness to be linearly related to species range and body size. Species described earlier likely have higher trait completeness because of their extensive research histories. I anticipate that trait completeness correlates with research effort, as measured by frequency of publications and species occurrence records.

Furthermore, I anticipated that endemic and threatened species are likely to have lower trait completeness than well-known and least threatened species. I hope this analysis will offer insights into the availability of life-history trait data for Philippine biodiversity, aiming to guide ongoing and future research while supporting conservation strategies rooted in trait-based ecology and management. Moreover, I contend that the emerging patterns are not unique to the Philippines or terrestrial vertebrates but are likely mirrored across other developing and megadiverse countries and other taxonomic groups, thereby extending the relevance and significance of this work to broader global biodiversity contexts.

## Results

### Trait coverage and completeness

**Figure 1:**
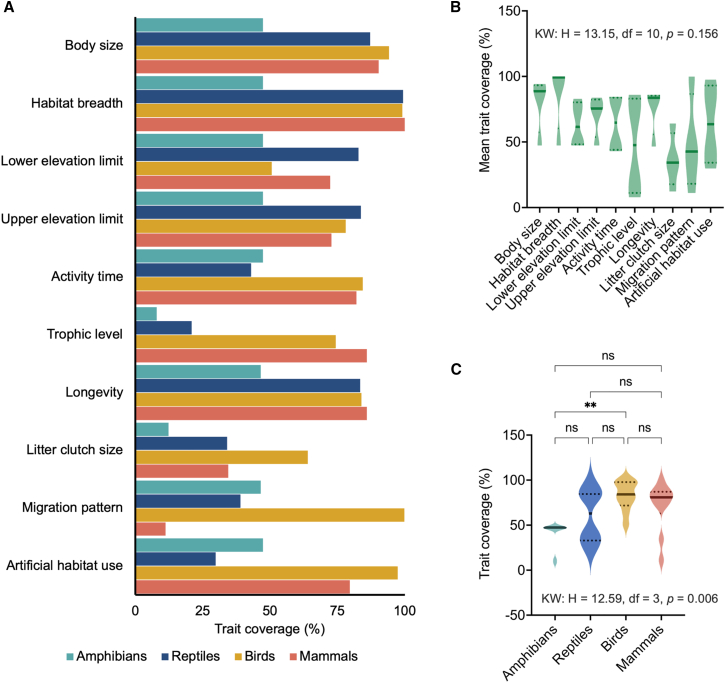
Summary and comparison of trait coverage among Philippine terrestrial vertebrates Trait coverage by trait type (A) and violin plots illustrating the coverage distribution by trait type (B) and vertebrate classes (C). Solid lines indicate the median values in the violin plots, with quartile values indicated by dotted lines. Significance was determined using the Kruskal-Wallis test and Dunn’s pairwise comparison. Note: ∗∗*p* < 0.05, ∗∗∗*p* < 0.01, ∗∗∗∗*p* < 0.001.

Trait data coverage varied significantly among terrestrial vertebrate groups (Kruskal-Wallis test: H = 12.44, df = 3, *p* = 0.006; Figures 1A and 1B). The trait sampling coverage was highest for birds (median = 84%, 95% confidence interval [CI] = 64%–99%) and mammals (median = 81%, 95% CI = 34%–90%) but lower for reptiles (median = 63%, 95% CI = 30%–87%) and amphibians (median = 47%, 95% CI = 12%–47%). However, pairwise comparisons showed that only birds and amphibians differed significantly in terms of trait coverage (Dunn’s test: *p =* 0.0045) (Figure 1C). Coverage did not vary considerably among traits (H = 13.15, df = 9, *p* = 0.156), although habitat breadth (median = 99%, 95% CI = 47%–94%) and body size (median = 89%, 95% CI = 47%–94%) showed the highest sampling effort (Figure 1C).

**Figure 2:**
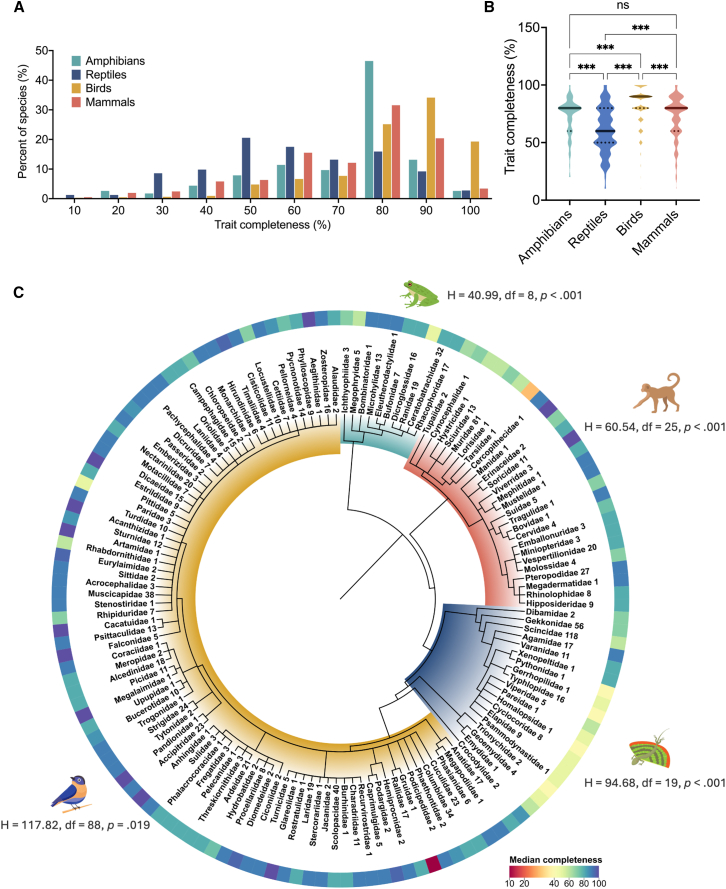
Summary and comparison of trait completeness of Philippine terrestrial vertebrates Frequency distribution of trait completeness (A) and violin plots illustrating the completeness distribution by vertebrate class (B). The solid lines indicate the median values in the violin plots, with quartile values indicated by dotted lines. (C) Comparison of median trait completeness across vertebrate classes at the family level. The number next to the family name indicates the number of species within the group used to compute the median values. Significance was determined using the Kruskal-Wallis test and Dunn’s pairwise comparison. Note: ∗∗*p* < 0.05, ∗∗∗*p* < 0.01, ∗∗∗∗*p* < 0.001.

The completeness distribution was generally left-skewed across all classes, with higher skewness observed among birds (−1.401) and amphibians (−1.181) than among mammals (−0.947) and reptiles (−0.099) (Figure 2A). Birds had the highest data completeness, with most species showing 80%–90% completeness, and at least 20% of the bird species had complete trait data, compared to less than 5% of other taxa. Mammals also exhibited relatively high completeness, with most species falling within the 70%–90% completeness range. While amphibians showed a bimodal pattern, at least 46% had 80% completeness, but only 3% had complete traits. In contrast, reptiles had the lowest trait completeness, peaking at approximately 50%, with most species having below 70%. Statistically, trait completeness differed significantly among classes (Kruskal-Wallis test: H = 298.7, df = 3, *p* < 0.0001), with birds exhibiting the highest completeness (median = 90%, 95% CI = 80%–90%) and reptiles the lowest (median = 60% [95% CI: 60-60%]) (Figure 2B). A pairwise comparison of trait completeness further showed significant differences among the groups, except between amphibians and mammals (Figure 2B). Subsequently, the analysis revealed that completeness was nonrandomly distributed across families in all classes (Figure 2C).

### Congruences in trait availability

**Figure 3:**
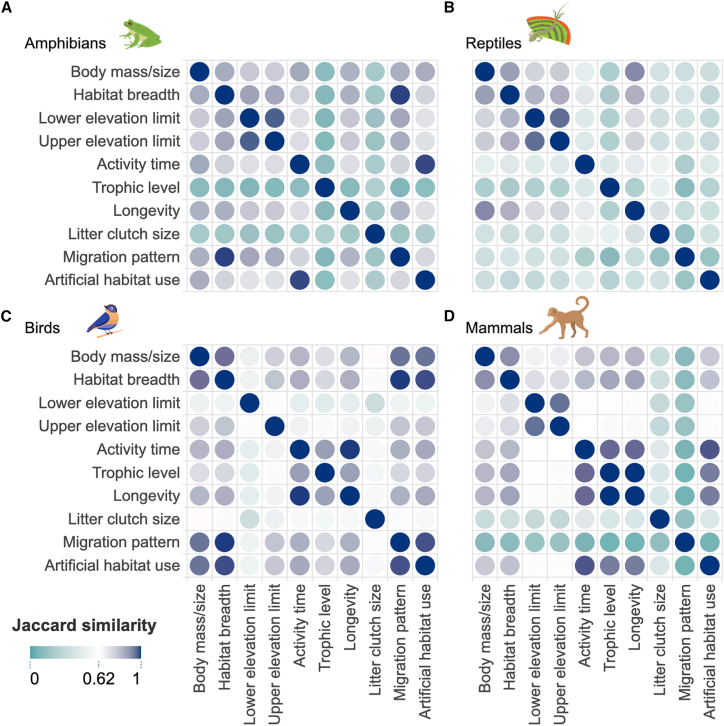
Congruence within-class similarities in trait availability Trait congruence based on Jaccard's index (*J*) across vertebrate classes classes: (A) amphibians, (B) reptiles, (C) birds, and (D) mammals. Tiles with darker colors indicate higher within-class similarity.

Within-class trait congruence showed that mammals exhibited the highest frequency of similar traits compared to other classes (Figure 3D). Trophic levels and activity time (*J*' = 0.955) showed a higher within-class congruence pattern (*J*' = 0.903), as did habitat breadth and body size. Other high within-class congruence in trait coverage included artificial habitat use and activity time (*J*' = 0.976), generation length and activity time (*J*' = 0.955), and artificial habitat use (*J*' = 0.932) alone. Activity time, artificial habitat use (*J*' = 0.988), migration pattern, and habitat breadth (*J*' = 0.991) exhibited the highest within-class coverage among amphibians. In reptiles, only longevity and body size (*J*' = 0.911) showed a notable congruence in trait availability. For birds, habitat breadth, body size (*J*' = 0.997), and migration patterns (*J*' = 0.943) demonstrated strong trait congruence (Figure 3).

### Correlates and differences in trait completeness

**Figure 4:**
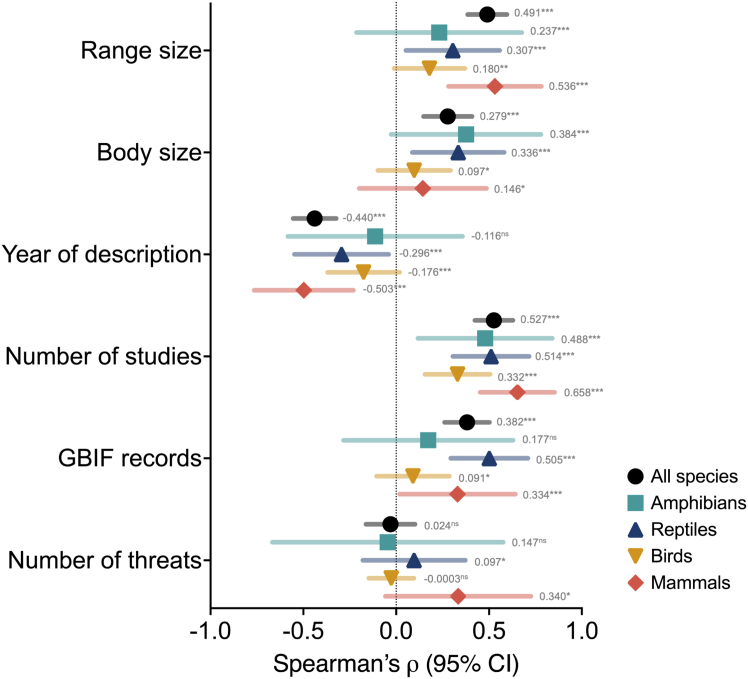
Relationship between body size and research effort on trait completeness Summary of Spearman’s ρ correlation between trait completeness and body size, year of description, number of studies, and number of GBIF occurrence records. Note: ns = not significant, ∗*p* < 0.05, ∗∗*p* < 0.01, ∗∗∗*p* < 0.001.

Trait completeness varied significantly and was related to multiple factors within the different taxonomic classes. Body size and species range were positively correlated with trait completeness in all taxonomic classes (Figure 4). While the year of species description was negatively correlated with trait completeness, indicating that species described earlier generally had higher trait completeness, this relationship was significant for all classes, except for amphibians. Research efforts based on Google Scholar hits have revealed a significant positive relationship between trait completeness and all taxonomic groups. Similarly, species with a higher number of Global Biodiversity Information Facility (GBIF) occurrence records exhibited significantly greater trait completeness; however, this was only evident among reptiles, birds, and mammals (Figure 4). Finally, the number of documented threat types showed a significant positive correlation with trait completeness in mammals and reptiles.

**Figure 5:**
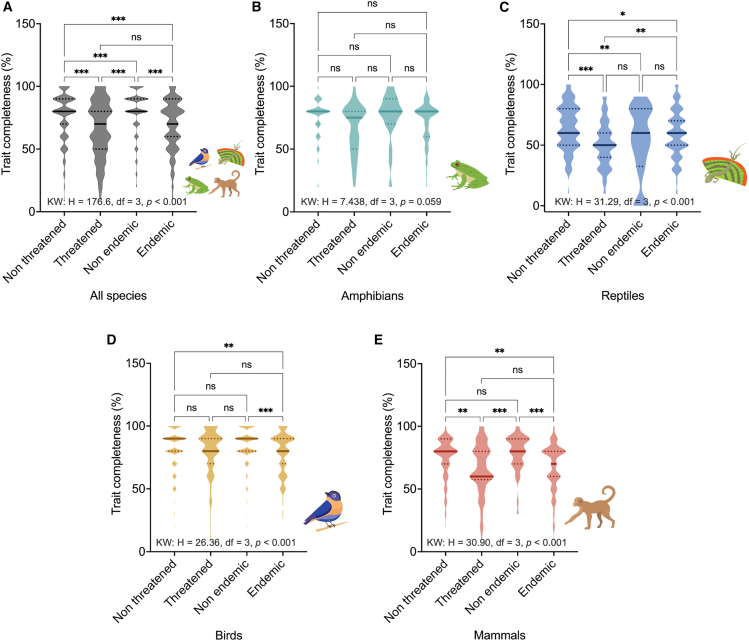
Difference in trait completeness among classes Violin plots illustrating the completeness distribution by conservation status and country endemism across vertebrate classes: (A) all groups, (B) amphibians, (C) reptiles, (D) birds, and (E) mammals. Solid lines indicate the median values in the violin plots, with quartile values indicated by dotted lines. Significance was determined using the Kruskal-Wallis test and Dunn’s pairwise comparison. Note: ∗∗*p* < 0.05, ∗∗∗*p* < 0.01, ∗∗∗∗*p* < 0.001.

Consequently, patterns of trait completeness varied between conservation status, endemism, and classes. Broadly, threatened and endemic species exhibited significantly lower trait completeness than non-threatened and more widespread species (Figure 5A). Specifically, no significant differences were observed among amphibians, regardless of their conservation status or endemism. Threatened reptiles and mammals exhibited significantly lower trait completeness compared to their non-threatened counterparts. On the other hand, differences related to endemism are evident only in birds and in mammals. Interestingly, reptiles were the only group in which conservation status and endemism interacted significantly, with threatened species exhibiting lower trait completeness than endemic species (Figures 5B–5E).

## Discussion

Trait-based conservation is widely adopted in the recent decades, which allows identifying priority populations based on morphological, ecological, behavioral, and physiological traits contributing to their vulnerability to environmental changes and threats.^43^^,^^54^^,^^55^ Yet, gaps on trait completeness remain a challenge. The current analysis showed significant differences in trait coverage and completeness among Philippine terrestrial vertebrates, a pattern consistent with a previous global trait assessment.^39^ However, apparent variations across groups linked to ecological status and research attention were also observed in this study, providing a more context-specific basis for conservation efforts in the Philippines. Limited trait data in developing countries, such as the Philippines, hamper comprehensive trait-based conservation, as many species lack the key life-history information. Trait coverage for terrestrial vertebrates is uneven, with birds and mammals having more data, whereas reptiles and amphibians are notably underrepresented. This is consistent with several prior macroecological studies and patterns observed in species discovery, which often focus more on vertebrate endotherms (mammals and birds) and relatively neglect reptiles and amphibians in ecological studies.^56^^,^^57^^,^^58^^,^^59^^,^^60^ These disparities across classes stem from historical research biases, as avian and mammalian species have received more attention owing to their detectability, ease of sampling, various biological attributes, and higher public support and funding than those of herptiles.^56^^,^^60^^,^^61^^,^^62^^,^^63^^,^^64^ Conversely, reptiles and amphibians, often characterized by cryptic behaviors, restricted distributions, and lesser charisma, have historically been understudied, and their life-history traits are frequently underdocumented.^58^^,^^65^^,^^66^ Raunkiæran shortfall disproportionately affects ectotherms such as amphibians and reptiles, many of which are endemic and threatened. The lack of trait documentation for many species from these groups not only obscures their extinction risk but also excludes them from conservation prioritization schemes, effectively rendering them invisible in national policy frameworks.

Congruences in trait availability co-occur but vary among groups, likely because of the differences in relative ease of collecting standardized data within certain classes. Traits such as habitat breadth and body size are widely observed within the same class since they can be co-collected or inferred, widely observable, and easily measurable.^67^ Body size can be measured during field sampling and collection or using museum specimens,^68^^,^^69^ while habitat breadth can be measured by the range and extent of habitat types a species occupies, as determined through field surveys and occurrence data. High within-class congruence indicates that trait data are relatively complete and uniformly available across species, thereby enhancing the reliability of downstream trait-based analyses and making them highly useful for addressing data gaps in trait data applications in ecology and evolution. When closely related species are available, imputation techniques can leverage shared traits to fill in the missing information more reliably.^70^^,^^71^ Furthermore, certain traits can act as proxies for others, meaning that a well-documented and easily measurable trait may provide strong indications of another less frequently observed trait. This cross-representation expands the analytical potential of the existing datasets. Conversely, low similarity in other trait groups highlights inconsistencies in trait representation, which may signal knowledge gaps or data biases that could affect inferences for conservation decisions in the future.

Trait completeness varied significantly across families within classes, indicating that trait availability is phylogenetically dependent. Well-represented families, particularly among birds and mammals, generally exhibit higher trait data completeness, which has important implications for imputing missing data within broadly represented groups in the study. Most importantly, species body size and range emerged as significant factors related to trait completeness, with larger-bodied and more widely distributed species generally exhibiting higher completeness across all classes, except for that of birds. Larger species are often more visible, easier to study, and more likely to be the focus of ecological and conservation research (i.e., due to their higher vulnerability to threats), which can explain their higher trait completeness.^60^^,^^72^ Yet, there is an exception among birds, which may reflect the more extensive and standardized ornithological databases that maintain comprehensive data, even for smaller species. For instance, AVONET, a global collaborative initiative, encompasses up-to-date, vast, and comprehensive functional trait datasets for birds, including six ecological variables, 11 continuous morphological traits, and data on range size and geographical distribution.^73^ Although there are global databases for mammals, such as PHYLACINE^74^ and PanTHERIA,^75^ and recently established databases, such as ReptTraits^76^ and AmphiBIO^77^ for reptiles and amphibians, respectively, many Philippine species remain underrepresented in these databases.

Conservation status and country endemism were related to trait completeness, with threatened and non-endemic species, particularly endotherms, showing lower trait completeness than their more widespread and non-threatened counterparts. Endemic species exhibit lower trait completeness and coverage due to their restricted geographic ranges, which limit data collection and inclusion in broad ecological surveys. Their occurrence in remote, specialized, or understudied habitats, coupled with their rarity or threat status, further constrains trait measurements. In contrast, non-endemic species and those with broader geographical ranges are often distributed across multiple regions, countries, or habitat types. This enables researchers to collect and infer trait information from diverse contexts and integrate these data into global biodiversity repositories, thereby increasing their overall trait completeness. This bias, favoring accessible and well-known species, results in underrepresentation of species needing conservation attention in trait databases.^39^^,^^43^^,^^78^ This imbalance further risks skewing conservation strategies toward well-documented groups, potentially neglecting others that may be equally or even more at risk.

The relationship between the year of species description and trait completeness suggests that earlier described species are generally better studied and more thoroughly understood, likely because they have been part of the scientific record for longer, attracting sustained research attention and repeated data collection.^68^^,^^73^^,^^74^^,^^79^ In contrast, recently described species often remain poorly studied, with limited trait information available due to shorter research histories, restricted sampling opportunities, and fewer museum collections.^64^^,^^80^^,^^81^ The poor coverage and completeness in more recently described species is concerning for recently described species that are already threatened, as incomplete knowledge of their ecological and biological characteristics hampers accurate risk assessments and may delay the development of effective conservation strategies.^7^^,^^60^ Additionally, species with extensive GBIF records are typically those that have been widely sampled from accessible areas or those that are easily detectable,^82^ leading to better trait documentation.^46^ The observed gap further suggests that targeted conservation research, especially for species with limited studies or occurrence records, is essential for improving trait data coverage, closing existing data gaps, and ensuring that conservation efforts are informed by comprehensive species information.^41^^,^^51^ Subsequently, I observed a positive correlation between the number of documented threats and trait completeness but only for mammals and reptiles. Species with more complete trait information are often better studied, increasing the likelihood of identifying and recording multiple threats. Conversely, species with sparse trait data may also have fewer documented threats, not necessarily because they face less risk but because of limited research attention. This pattern further highlights the uneven nature of biodiversity knowledge, suggesting that threat assessments may underestimate the risks for poorly studied species.

Life-history traits serve as a valuable common currency in advancing modern ecology and conservation, enabling macroecological analyses relevant to providing insights into how species can survive, adapt, or what populations are likely to be at risk in a changing environment.^83^ Developing countries, such as the Philippines, contain many endemic and threatened species that remain poorly studied in terms of conservation-relevant traits.^46^ Consequently, analyses of biodiversity patterns and environmental sensitivity often rely on incomplete data, hampering our understanding of species extinction drivers.^11^^,^^39^^,^^43^^,^^84^ These data gaps may significantly hinder the development of effective and efficient conservation planning and management strategies.^12^^,^^43^^,^^85^^,^^86^ When data primarily exist for charismatic, economically significant, or well-distributed species, conservation priorities may favor these better-studied taxa over those needing protection.^21^^,^^87^^,^^88^ This can result in resource allocation that overlooks data-poor species, particularly rare or endemic species facing extinction risk.^89^^,^^90^^,^^91^

In biodiversity hotspots, particularly in nations with limited funding for comprehensive conservation efforts, the absence of robust life-history data can lead to conservation strategies that overlook species that are inherently more vulnerable owing to their ecological characteristics, such as slow reproductive rates or high habitat specialization.^41^^,^^42^^,^^43^ These gaps can cascade through conservation assessments, leading to systematic underrepresentation of key traits or taxa in global evaluations. Ultimately, such distortions reduce the effectiveness of conservation actions and may exacerbate biodiversity loss by overlooking the most imperilled components of ecosystems. Species risk assessments, such as those used in IUCN Red List evaluations, rely heavily on life-history traits, and data gaps can result in misclassification of species conservation statuses.^92^^,^^93^^,^^94^ These further skews the national conservation agenda and reduces the efficiency of resource allocation, which is a critical concern for developing countries with limited conservation budgets.

**Figure 6:**
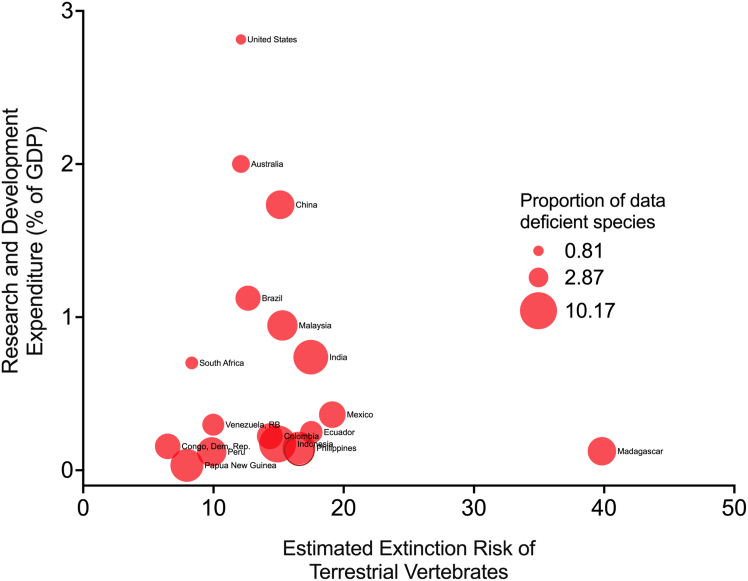
Broader socioecological and economic drivers Research funding and expenditure trends in megadiverse countries highlight disparities linked to extinction risks and the proportion of data-deficient species. Economically strong nations (e.g., the United States, Australia, Brazil, China, and Malaysia) generally allocate higher research budgets, whereas less affluent but equally biodiverse countries remain underfunded. Note: data for research and development expenditure (% of GDP) were averaged from 2000 to 2023, extracted from the World Bank Open Data.^101^

Gaps in trait data reflect broader systemic gaps in Philippine biodiversity research, with multiple factors limiting our understanding of the country’s natural history.^46^ Megadiverse countries host thousands of species, making it challenging to collect comprehensive trait data. Despite high biodiversity, limited resources are allocated to biodiversity research and conservation, which are often considered secondary to human welfare needs.^95^^,^^96^ This underfunding constrains data collection and assessments.^97^^,^^98^^,^^99^ Compared among megadiverse countries, the Philippines ranks 14^th^ in research expenditure as a percentage of GDP, despite its high extinction risk and data-deficient species (Figure 6). This pattern is observed across megadiverse countries, where those with higher extinction risks allocate research expenditures poorly as a percentage of GDP. Megadiverse countries with stronger economies, such as the United States, Australia, Brazil, China, and Malaysia, allocate higher research budgets^100^ (Figure 6). In particular, a megadiverse country with a similar rate of extinction risk as China has multiple folds in its biodiversity research investment, focusing on management and database development in recent decades as part of its commitment to the environment and nature.^102^ This inequitable allocation of funds to support research further hinders conservation, as regions with the highest species diversity lack the financial capacity to study their ecosystems.^96^^,^^103^

While proper permitting is necessary for biodiversity studies and collection,^104^ I argue that bureaucratic red tape and complex permitting processes exacerbate geographic and taxonomic biases in trait datasets, hindering data collection for endemic and threatened species in protected and remote habitats, particularly in developing nations.^104^^,^^105^^,^^106^^,^^107^^,^^108^ Streamlining permitting systems and establishing equitable collaboration with local communities and agencies offer opportunities to improve data coverage and strengthen the availability of traits. Furthermore, lacking research facilities and expertise further constrains life-history trait database development.^39^^,^^41^^,^^51^

### Moving forward

Raunkiæran shortfall would impede efficient analysis for practical conservation. Without a robust dataset for trait data, predictive models become unreliable, limiting species risk forecasting.^11^^,^^12^^,^^109^ This gap hinders conservation prioritization and obscures trait patterns linked to extinction risk, which are crucial for developing strategies.^109^ Conservation efforts thus favor well-studied species while overlooking taxa with limited trait data. This further restricts the application of conservation tools, such as habitat restoration and protected area design, which mostly rely on understanding species ecological roles and environmental requirements.^42^^,^^83^ A lack of harmonized trait data across taxonomic groups impedes comparative analyses and limits the generalization and coordination of cross-species conservation strategies. In contrast, species with complete trait profiles benefit only if these traits capture threat-relevant information, highlighting the importance of having significant traits.

In megadiverse but data-deficient regions, the challenges in species trait availability are even more pronounced, slowing their inclusion in global conservation priorities and limiting access to international support and funding mechanisms. Filling gaps in trait data is a difficult challenge for developing megadiverse countries such as the Philippines, which often face similar or greater economic constraints that hinder the mobilization of critical biological data.^51^ These challenges are likely mirrored in other biodiverse nations with limited resources, underscoring the need to adopt multiple strategies to address these data deficiencies. To tackle the persistent Raunkiæran shortfall and advance trait-based conservation, promoting equitable and collaborative research that can boost access to expertise and funding for trait-based studies is needed (Table 2). Partnerships between institutions (for example, South-South or North-South) to mobilize trait data from collections, following FAIR principles, can help address gaps in trait data in developing megadiverse countries. Investing in establishing centralized and open-access trait databases can ensure that the collected data are accessible and widely used to advance conservation efforts.^41^^,^^55^ Citizen science initiatives can rapidly increase trait data, particularly for well-known taxa.^110^ Machine learning and other predictive approaches can estimate missing life-history traits based on phylogenetic and ecological proxies.^39^ Regional collaborations offer promising pathways for harmonizing trait data standards, sharing imputation methods, and co-developing tools tailored to meet regional needs. These methods, primarily when transparently reported, can provide interim solutions when more data are collected. International platforms such as GBIF and GeoBON can serve as hubs to facilitate these efforts, enabling megadiverse nations to overcome data shortfalls collectively and strengthen their participation in global biodiversity science and conservation efforts.^51^^,^^111^^,^^112^ More importantly, addressing the deficiency in ecological research within the country, likely attributable to limited capacity, insufficient funding, and a lack of collaborative culture, represents a crucial step forward. This initiative is essential for advancing the significance of trait-based ecology in the Philippines and other megadiverse regions, thereby addressing the gaps in biodiversity knowledge.^54^^,^^113^

**Table 2:** Suggested ways and mechanisms to improve trait data-sharing in developing nations facing challenges and difficulties

1. Collaborate with global trait initiatives by linking local datasets with large-scale repositories and consortia, which reduces duplication, fills gaps efficiently, and ensures that local data contribute to broader conservation efforts. 2. Prioritize and standardize essential traits such as body size, reproductive rates, and habitat breadth, using common typologies and metadata standards to ensure that data remain comparable and interoperable across studies and analysis. 3. Engage citizen scientists, students, and local communities through simple, low-cost protocols for measuring observable traits, which expand coverage across remote regions and reduce the burden on limited research budgets. 4. Digitize and re-use specimens in museums, herbaria, and universities by extracting morphological and ecological trait data from existing collections, providing a cost-effective way to expand trait completeness without new fieldwork. 5. Promote FAIR data sharing through incentives and streamlined processes, including recognition, co-authorship, small-targeted grants, and national biodiversity alliances that minimize bureaucratic hurdles and make trait data openly available.

Lastly, addressing the Raunkiæran shortfall is not merely an academic exercise; it is fundamental to ensure that conservation decisions in megadiverse countries, such as the Philippines, are inclusive, data-informed, and capable of preventing extinction. Despite challenges ranging from funding constraints to limited accessibility of study species, scientific expeditions and fieldwork remain vital for filling data gaps, generating new trait information, and ensuring continuous monitoring, thereby improving trait availability and reducing knowledge shortfalls, particularly for ecologically significant groups. Without trait data, the species in need of protection may remain overlooked in a system designed to save them.

In conclusion, the limited availability of life-history traits in developing megadiverse countries poses a significant barrier to practical trait-based conservation efforts. However, recognizing these challenges as opportunities can empower these nations to harness trait data for more informed biodiversity management and policymaking.

### Limitations of the study

While the current analysis provides fundamental information on the current extent of the Raunkiæran shortfall in Philippine biodiversity, as a case study for a megadiverse country, I acknowledge some key caveats in the current work. A critical limitation of this study was its reliance on open-source global IUCN assessments, which may not always reflect the most current or comprehensive information.^114^^,^^115^ Some species lack recent standardized updates or thorough evaluations, and localized threats are often underrepresented in global assessments.^94^ For instance, populations categorized as “Least Concern” globally may, in reality, face greater pressure at the local scale,^116^ making them highly threatened within specific regions despite their seemingly secure global status. In addition, the reliance on secondary databases, which are valuable for broader assessments, may exclude locally sourced and unpublished data. For example, in the historically conflicted southern Philippines, a large proportion of species occurrence data and their associated metadata were inaccessible until recent data mobilization efforts.^53^^,^^95^^,^^99^ A similar pattern is likely actual for species trait data across the Philippines, much of which remains undigitized in museums or only exists in gray literature, subsequently underutilized in biodiversity analyses.^44^^,^^51^^,^^53^ The non-inclusion of empirical field data, museum specimen measurements, and indigenous ecological knowledge limits the completeness and contextual relevance of trait datasets, particularly for poorly studied and endemic species in countries like the Philippines. Moreover, this study focused only on fundamental traits that are broadly available across taxa. While this allows for a general comparison, it excludes taxon-specific traits, such as wing morphology in bats or reproductive strategies in amphibians, which could be critical for accurately assessing extinction and threat vulnerability.

Additionally, research efforts were estimated using proxies such as publication counts and GBIF occurrence records, which may not reflect the true extent or focus of biodiversity research, such as the actual funding allocation for biodiversity research. These indicators often favor well-documented species and overlook contributions from local institutions or gray literature. Importantly, this study did not directly test the influence of traits on extinction risk. Although trait availability and bias patterns have been described, their predictive value for extinction vulnerability remains unexplored. Finally, future research incorporating field-based trait data, more refined effort metrics, and explicit extinction risk modeling would enhance the utility of trait-based approaches for conservation planning in megadiverse but data-limited regions worldwide.

## Resource availability

### Lead contact

Requests for further information and resources should be directed to and will be fulfilled by lead contact, Krizler C. Tanalgo (tkrizler@gmail.com).

### Materials availability

No new reagents were used in this study.

### Data and code availability

• Links to data repositories are in the key resources table.
• No custom code has been developed for this study. All statistical analyses and visualizations were performed using standard data analysis and visualization software.
• Any additional information required to reanalyze the data reported in this article is available from the lead contact upon request.

## Acknowledgments

I thank the editor and two anonymous reviewers for their insightful and constructive comments and suggestions, which improved the quality of the manuscript.

## Author contributions

This is a sole-authored work. The author conceived, designed, conducted, analyzed, and wrote the manuscript.

## Declaration of interests

The author declares no competing interests.

## Declaration of generative AI and AI-assisted technologies in the writing process

The author utilized Paperpal during the preparation of this manuscript to improve clarity, coherence, and structure. All revisions were carefully reviewed and refined by the author, who accepted full responsibility for the final version of the manuscript.

## STAR★Methods

### Key resources table

**Table:** 

REAGENT or RESOURCE	SOURCE	IDENTIFIER
**Deposited data**		

Species list	IUCN Red List (2025-1)^117^	https://www.iucnredlist.org/
Species trait (11 trait dataset)	Etard et al.^118^	https://figshare.com/articles/dataset/Global_gaps_in_terrestrial_vertebrate_trait_data/10075421
Elevation range	IUCN Red List (2025-1)^117^	https://www.iucnredlist.org/
Habitat breadth	IUCN Red List (2025-1)^117^	https://www.iucnredlist.org/
Migration pattern	IUCN Red List (2025-1)^117^	https://www.iucnredlist.org/
Species conservation status	IUCN Red List (2025-1)^117^	https://www.iucnredlist.org/
Species endemism	IUCN Red List (2025-1)^117^	https://www.iucnredlist.org/
Number of studies	Google Scholar	https://scholar.google.com/
Species occurrence records	Global Biodiversity Information Facility (GBIF)^119^	https://www.gbif.org/
Year of description	Catalog of Life (CoL)^120^	https://www.checklistbank.org/tools/name-match
Research and development expenditure (% of GDP)	World Bank Open Data^101^	https://data.worldbank.org/indicator/GB.XPD.RSDV.GD.ZS

**Software and algorithms**		

JAMOVI version 2.6	The Jamovi Team^121^	https://www.jamovi.org/
GeoCAT	Bachman et al.^122^	https://geocat.iucnredlist.org/
PAST version 5.0	Hammer et al.^123^	https://www.nhm.uio.no/english/research/resources/past/
Scimago Graphica version 1.0.50	Hassan-Montero^124^	https://www.graphica.app/
GraphPad Prism version 9	GraphPad Prism^125^	https://www.graphpad.com/features

### Method details

#### Trait compilation and assembly

The species list for extant amphibians, reptiles, birds, and mammals occurring in the Philippines was extracted from the latest IUCN Red List database (2025-1) (https://www.iucnredlist.org/), including only species in terrestrial systems and inland freshwater habitats, excluding domesticated species.^117^ Downloaded files of the species list were cleaned and organised according to class. The final analysis included 114 amphibians (9%), 323 reptiles (26%), 648 birds (49%), and 206 mammals (16%). Ten life-history trait datasets standard to all classes were primarily obtained and assembled from global trait datasets for terrestrial vertebrates from Etard et al.^39^^,^^118^ To improve the dataset, I added the generation length data for amphibians and reptiles obtained from Mancini et al.^33^ and the elevation range and migratory pattern from the IUCN Red List.^117^ Species habitat breadth was recomputed based on the latest IUCN dataset. Correlated and overlapping traits were consolidated to minimise redundancy, and the most complete and informative trait set was selected for subsequent analysis. Trait completeness rates and coverage were calculated for each class. In this context, the completeness rate is defined as the number of traits with available data divided by the total number of traits assessed for a given species. Trait coverage was calculated as the proportion of species with data for a given trait (i.e., the number of species with data for that trait divided by the total number of species with data).^39^

#### Variables related to trait completeness

Following my initial hypothesis, species body mass or size, extinction risk, country endemism, species description year, research effort, and number of threats were tested for their relationships with trait completeness. Species body mass (in grams) and size were obtained from Etard et al.^39^ A simplistic binary classification of extinction risk was subsequently applied to differentiate between species based on their probability of extinction or persistence.^126^ To determine the geographic range of all species, both the Extent of Occurrence (EOO) and Area of Occupancy (AOO) were calculated using GeoCAT.^122^ Georeferenced occurrence records were obtained from the GBIF and iNaturalist, and spatial filtering was applied to remove duplicates and outliers.^127^ The EOO was estimated as the minimum convex polygon (MCP) encompassing all known, inferred, or projected occurrence points, representing the overall distribution range of the species. In contrast, the AOO was derived following the IUCN guidelines by overlaying a 2 km × 2 km grid across the occurrence data and summing the area of the occupied grid cells.^122^

Species considered ‘Critically Endangered,’ ‘Endangered,’ or ‘Vulnerable’ were grouped under ‘Threatened.’ Conversely, ‘Near Threatened’, ‘Least Concern’, or ‘Lower Risk’ species were assigned to the ‘Non-threatened’ category. Species classified as ‘Data Deficient’ or ‘Not Evaluated’ were also included in the ‘Threatened’ group based on the assumption that they are more likely to have limited distributions and smaller populations, making them more susceptible to threats and local population decline.^89^^,^^90^^,^^128^ Country endemism was defined as a species occurring exclusively within the Philippines throughout its entire life-history.^91^ Species description years were derived based on the name-matching tool in the ChecklistBank (https://www.checklistbank.org/tools/name-match).^120^ Finally, research effort was measured based on Google Scholar hits and the number of occurrence records of species in the Philippines from the Global Biodiversity Information Facility (GBIF) (https://www.gbif.org/) (see Tanalgo et al.^46^).

Finally, the number of threats was based on the level-1 threat classification in the IUCN Red List (version 3.2),^129^ which defined 11 major categories: (1) residential and commercial development, (2) agriculture and aquaculture, (3) energy production and mining, (4) transportation and service corridors, (5) biological resource use, (6) human intrusion and disturbance, (7) natural system modifications, (8) invasive and other problematic species, genes, and diseases, (9) pollution, (10) geological events, and (11) climate change and severe weather. For each species, I recorded whether a given threat type was reported (scored as 1) or absent (scored as 0) from the IUCN and additional literature, generating a matrix of up to 11 possible threats per species. From this, we derived the cumulative number of threats affecting each species and summarised the frequency of each threat category across classes.

### Quantification and statistical analysis

#### Statistical analysis

Before the analysis, necessary data treatments and tests were conducted, and each dataset was examined to ensure that it followed statistical assumptions. All continuous data were log_10_-transformed (x+1) prior to analysis. All data analyses were implemented in the open statistical software, Jamovi (v 2.6.).^121^ Data visualisation was performed using GraphPad Prism^125^ and SciMago Graphica.^124^ A presence-only Jaccard similarity analysis was performed using PAST software.^123^

The data did not conform to the normality assumption; therefore, a non-parametric Kruskal–Wallis test was employed to assess the differences in trait coverage and completeness across the classes. Post-hoc pairwise comparisons between classes were subsequently conducted using Dunn’s test. Subsequently, Jaccard’s similarity test was used to evaluate congruence in trait availability (i.e., presence or absence) within-class, across groups. Analysing within-class trait congruence allows quantifying how consistently traits are represented within each taxonomic group based on availability. Here, I evaluate whether species within a class (e.g., mammals, birds, reptiles, and amphibians) share similar trait information coverage or whether there are substantial gaps. The Jaccard index values ranged from 0 to 1, with values close to 1 indicating a higher similarity in the presence of traits within a class. Class-specific Spearman’s correlation tests were conducted to determine the congruence between species trait completeness and variables such as body mass or size, species description year, number of studies, GBIF occurrence, and number of threats. Consequently, trait completeness was compared among classes based on their ecological status (e.g., extinction risk and country endemism) using the Kruskal–Wallis test, followed by Dunn’s test for post-hoc pairwise comparisons.
